# Transaction of the Southern Surgical and Gynecological Association

**Published:** 1892-08

**Authors:** 


TRANSACTIONS OF THE SOUTHERN SURGICAL AND GYNECOLOGICAL ASSOCIATION.—Vol. IV. Fourth session, held at Richmond, Va., November 10, 11 and 12, 1891.

RARELY has an Association, even of much greater age, made a record equal to the one as is shown by this volume of Transactions. Organized a little less than five years ago, its success has been phenomenal, and to-day its members are recognized as among the leading lights of the profession on the American continent.

To Dr. W. E. B. Davis, of Rome, Ga., one of our most skilled abdominal surgeons, who was one of the original projectors, and who has been its Secretary since organization, is due much credit for the unremitting manner in which he has toiled for the success of this Society. C. E. J.

